# Cord Blood DNA Methylation and Large-for-Gestational-Age Birth: A Pilot Epigenome-Wide Study in the GROW Cohort

**DOI:** 10.3390/epigenomes10030051

**Published:** 2026-08-04

**Authors:** Xiaoyu Liang, Christopher Doumith, Dawn P. Misra, Vinod K. Misra

**Affiliations:** 1 Department of Epidemiology and Biostatistics, MSU College of Human Medicine, Michigan State University, 909 Wilson Road Room B645, East Lansing, MI 48824, USA; liangx20@msu.edu; 2 Children’s Hospital of Michigan, School of Medicine, Wayne State University, Detroit, MI 48201, USA; cdoumit1@hfhs.org; 3 Discipline of Pediatrics, School of Medicine, Central Michigan University, Mt. Pleasant, MI 48859, USA; vkmisra99@gmail.com; 4 Division of Genetic, Genomic, and Metabolic Disorders, Children’s Hospital of Michigan, Detroit, MI 48201, USA

**Keywords:** large for gestational age, DNA methylation, epigenome-wide association study, cord blood, fetal growth

## Abstract

**Background/Objectives:** Large-for-gestational-age (LGA) birth is associated with adverse perinatal outcomes and increased risk of metabolic disease later in life. Despite these risks, epigenetic studies of LGA remain limited, particularly those using umbilical cord blood DNA methylation (DNAm) as the tissue of interest. **Methods:** We conducted an epigenome-wide association study of cord blood DNAm in a nested case–control sample from the Gestational Regulators of Weight (GROW) cohort. The analysis included 28 term LGA infants and 63 term appropriate-for-gestational-age (AGA) controls. DNAm was measured using the Illumina HumanMethylation450 BeadChip. Epigenome-wide association analyses were performed with adjustment for residual principal components and estimated cord blood cell-type proportions. **Results:** No CpG sites reached statistical significance after false discovery rate correction. Twenty-four CpGs showed nominal associations with LGA status at *p*-value < 1.00 × 10^−4^, including five CpGs with absolute methylation differences greater than 0.05. Among annotated loci, the strongest interpretable signals included cg06750897 in *PBX1* (Δβ = 0.110, *p*-value = 1.70 × 10^−5^) and two nearby CpGs in *SMAD3*, cg23731272 (Δβ = 0.111, *p*-value = 4.31 × 10^−5^) and cg02486855 (Δβ = 0.109, *p*-value = 9.45 × 10^−5^). These two *SMAD3* CpGs showed concordant hypermethylation in LGA infants and formed a candidate regional methylation signal in exploratory targeted regional analysis. **Conclusions:** In this pilot study, LGA was associated with modest cord blood DNAm differences, although no CpG sites reached genome-wide significance. The strongest interpretable signals involved *PBX1* and *SMAD3*, suggesting candidate loci for further evaluation in larger studies of fetal overgrowth.

## 1. Introduction

The obesity epidemic in the United States continues to escalate [1]; consequently, nearly half of all current births in the United States occur among overweight or obese women [2]. One of the earliest adverse effects of maternal obesity is altered birth weight relative to gestational age. Infants whose birth weight falls between the 10th and 90th percentiles for gestational age are classified as appropriate-for-gestational-age (AGA), while infants born weighing more than the 90th percentile are classified as large-for-gestational-age (LGA). LGA infants are at an increased risk of complications during pregnancy and delivery [3] and may have an increased risk of stillbirth and neonatal death [4]. Additionally, LGA infants remain heavier throughout early childhood compared to their appropriate- and small-for-gestational age peers and are predisposed to developing obesity, metabolic syndromes, type II diabetes, and hypertension in later life [5,6,7,8,9].

Despite the clear clinical and public health risks for LGA both short-term and later in life, there has been little research on epigenetic pathways to LGA. Emerging data suggest that changes in DNA methylation (DNAm) may lead to alterations in metabolism and physiology of offspring [10,11]. Nearly all reports on DNAm and birth size have focused on small for gestational age at birth (reduced growth) as the outcome [12,13,14] or examined birth weight relative to gestational age across the continuum [15,16]. To our knowledge, only a few published studies have conducted epigenome-wide association studies (EWAS) examining LGA as a categorical outcome or increased fetal growth as a continuous outcome [16,17,18,19,20,21,22]. Recent studies further demonstrate both the biological relevance and the interpretive complexity of cord blood DNAm. In a large EWAS of 3116 mother–child pairs, maternal BMI was associated with cord blood DNAm at seven CpG sites, and methylation at *SP6* partially mediated the association between maternal BMI and childhood BMI [23]. A study of sorted cord and adult white blood cells showed that DNAm profiles are strongly dependent on cell type and developmental stage, underscoring the importance of accounting for cord blood cellular composition in EWAS [24]. In other neonatal populations, *LEP* promoter methylation in preterm cord blood was associated with inflammatory and growth-related measures, whereas cord blood DNAm at *CHD13* was associated with adiponectin levels at 10–14 years of age, further linking methylation at birth with later metabolic phenotypes [25,26]. However, these recent studies did not directly compare LGA and AGA infants, and evidence specific to LGA remains limited.

To contribute to the nascent literature on LGA and epigenetic pathways, we analyzed data from a nested case–control sample of 28 LGA infants and 63 AGA infants within the Gestational Regulators of Weight (GROW) study. We focused on DNAm measured in umbilical cord blood, which provides a measure of fetal DNAm patterns at birth and may capture systemic signals related to fetal growth. We hypothesized that LGA status would be associated with DNAm differences at loci involved in fetal growth, metabolic regulation, and immune/inflammatory signaling, potentially reflecting maternal metabolic exposures, fetal responses to the intrauterine environment, or both. We aimed to identify CpG sites and regional methylation signals for evaluation in larger studies.

## 2. Results

We analyzed cord blood DNAm in a nested case–control sample from the GROW cohort, including 28 term LGA infants and 63 term AGA control infants. After quality control, epigenome-wide analyses were conducted for 428,858 CpG sites. We first describe participant characteristics and then present the DNAm association with LGA status.

### EWAS of Cord Blood DNAm and LGA Status

Demographic and clinical characteristics by case–control status are shown in Table 1. Maternal and infant characteristics were similar between AGA and LGA infants, except that LGA infants were more likely to be born to overweight or obese mothers than AGA infants (75.0% vs. 42.9%, *p*-value = 0.006). Distributional assessments of the continuous variables are presented in Supplementary Figure S1. Birth weight was approximately normally distributed among AGA infants (Shapiro–Wilk *p*-value = 0.635) but departed from normality among LGA infants (*p*-value = 0.002). Gestational age at delivery showed no evidence of departure from normality in either the AGA or LGA group (*p*-values = 0.285 and 0.765, respectively). Accordingly, birth weight was compared using the Wilcoxon rank-sum test. As expected from the case–control definition, median birth weight was higher among LGA infants than AGA infants (4082.5 [IQR: 3983.8–4147.5] g vs. 3335.0 [IQR: 3192.5–3527.5] g; *p*-value < 0.0001). Mean gestational age at delivery was similar between the LGA and AGA groups (39.4 vs. 39.3 weeks; *p*-value = 0.762).

We then performed an EWAS to evaluate associations between cord blood DNAm and LGA status. The EWAS model adjusted for potential confounding using the top five residual principal components and estimated cord blood cell-type proportions. No CpG sites remained statistically significant after false discovery rate (FDR) correction. However, 24 CpGs showed nominal associations with LGA status at a *p*-value < 1.00 × 10^−4^ (Table 2).

**Figure 1:**
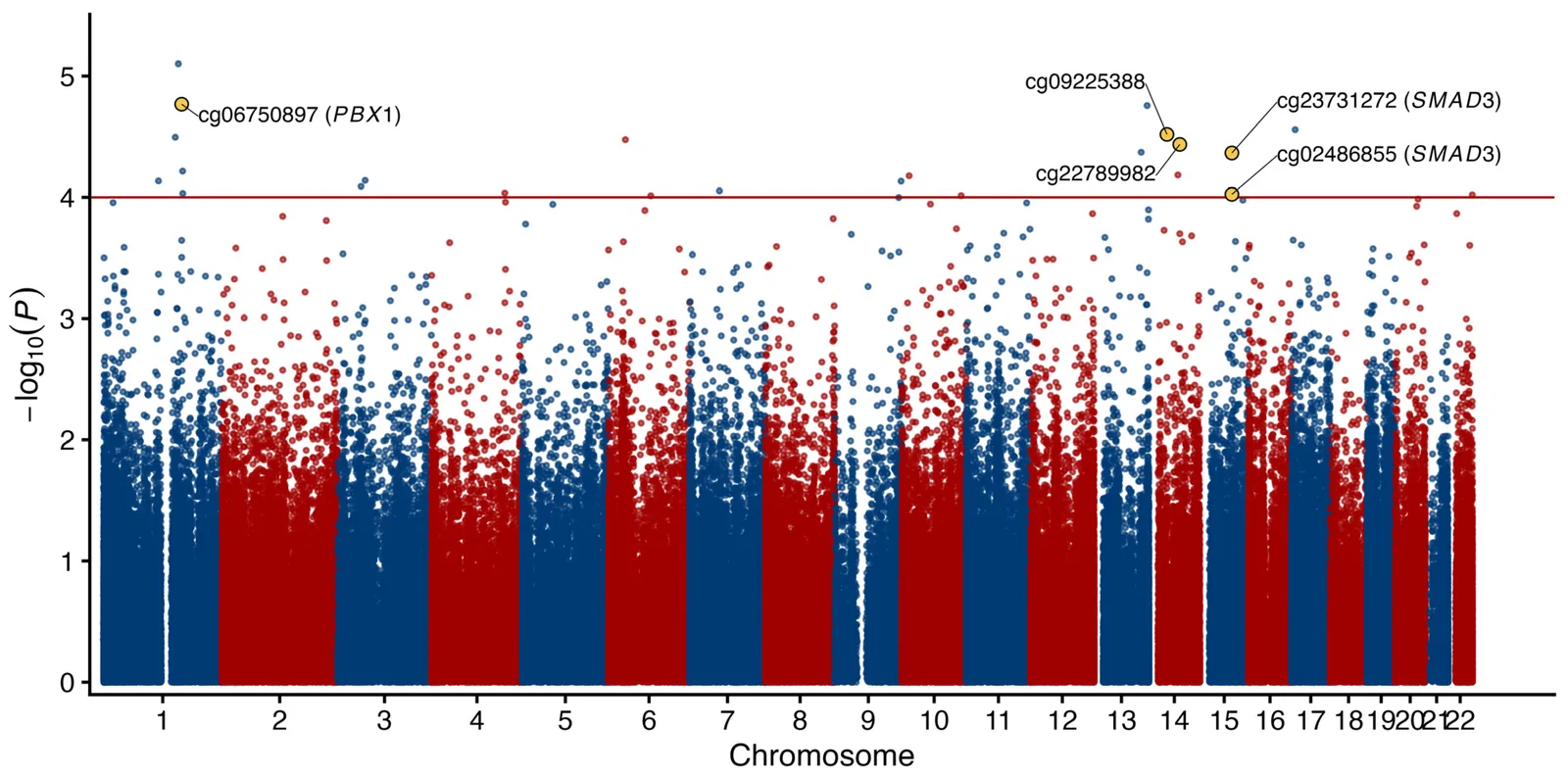
Manhattan plot of chromosomal locations of $-\log_{10}\left(p\right)$ for epigenome-wide association in 428,858 CpGs among the 91 samples. The red line represents the threshold 1.00 × 10^−4^. Yellow points indicate CpG sites with *p*-value < 1.00 × 10^−4^ and |Δβ| > 0.05.

**Figure 2:**
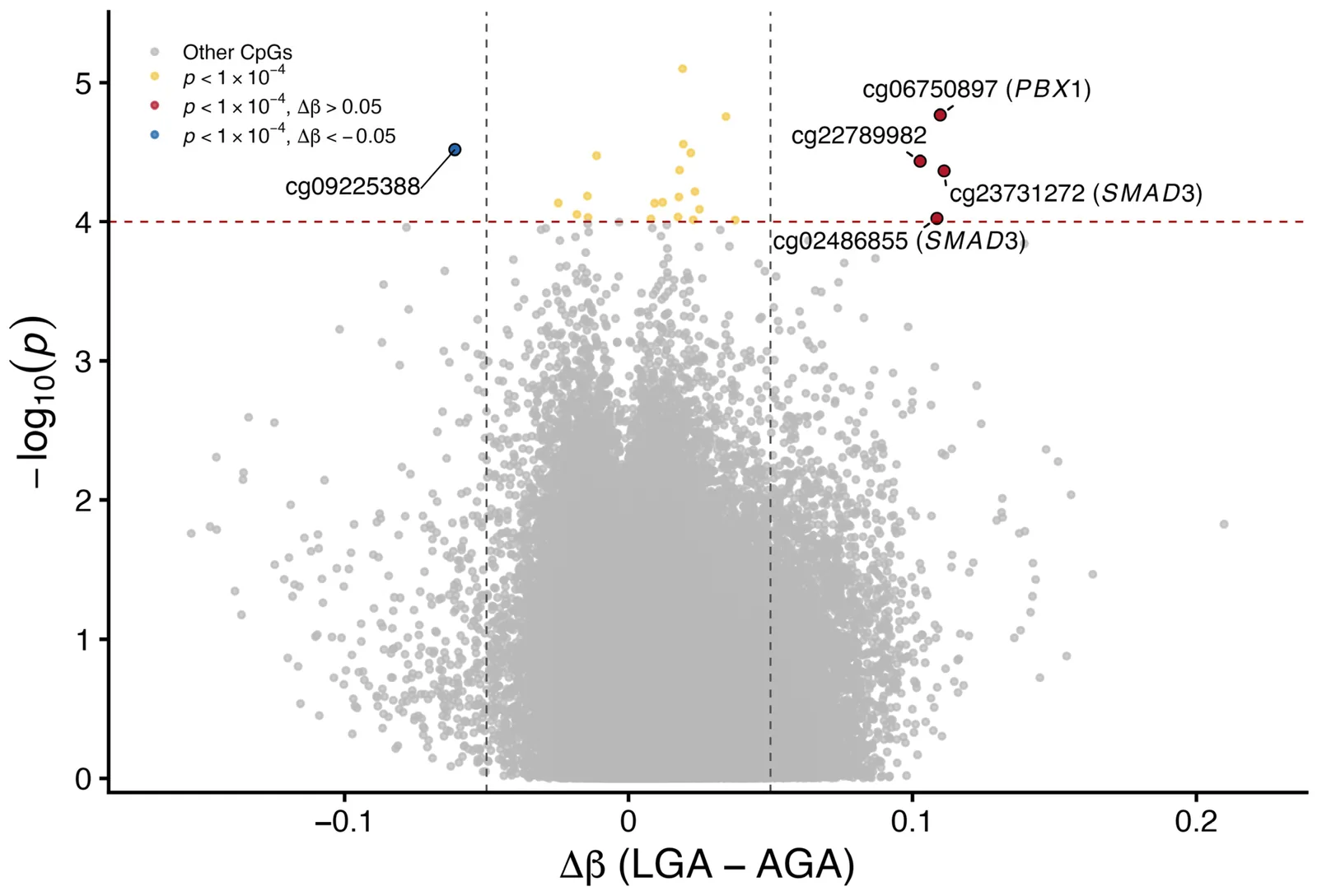
Volcano plot of cell-type adjusted EWAS results for LGA versus AGA infants. Each point represents one CpG site. The *x*-axis shows Δβ, calculated as the mean DNAm beta value in LGA minus AGA, and the *y*-axis shows −log10(*p*). Dashed lines indicate *p* < 1.00 × 10^−4^ and |Δβ| = 0.05. Highlighted and labeled CpGs met *p* < 1.00 × 10^−4^ and |Δβ| > 0.05. The model adjusted for the top five residual PCs and estimated cord blood cell-type proportions.

Figure 1 displays the Manhattan plot of −log10(*p*-value) across the genome for the association between LGA status and DNAm, with the 24 CpGs indicated above the red threshold line. Figure 2 shows the corresponding volcano plot, which displays both statistical evidence and methylation effect size, calculated as Δβ. Positive Δβ values indicate hypermethylation in LGA infants, whereas negative Δβ values indicate hypomethylation. Among the 24 nominally associated CpGs, 18 were hypermethylated and 6 were hypomethylated in LGA infants compared with AGA infants. The nominal *p*-values ranged from 7.92 × 10^−6^ to 9.72 × 10^−5^, and Δβ values ranged from −0.061 to 0.111.

**Figure 3:**
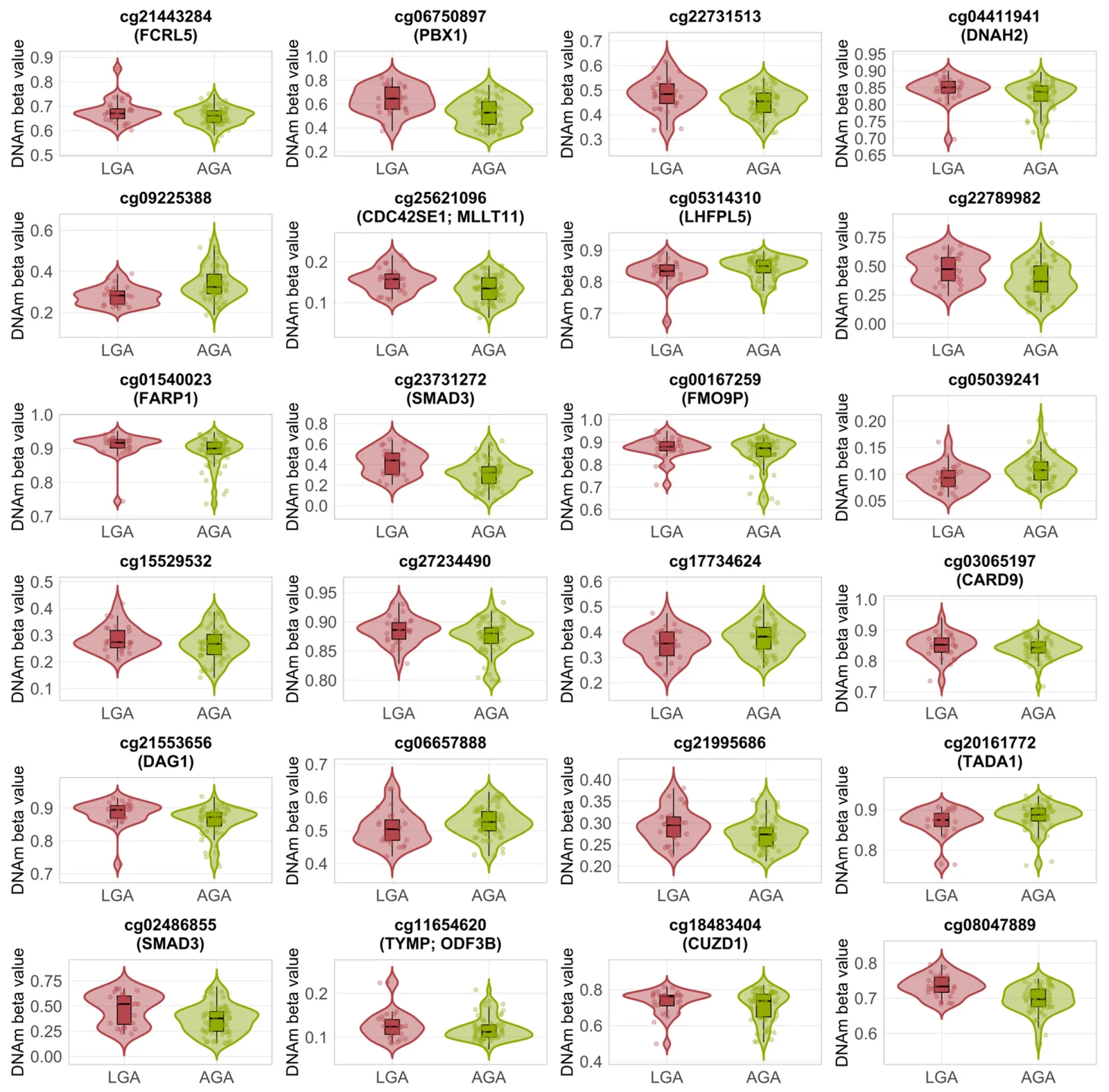
Violin plots of DNA methylation (DNAm) beta values for the 24 CpGs with nominal *p* < 1.00 × 10^−4^ for large-for-gestational-age (LGA) and appropriate-for-gestational-age (AGA) infants.

Five CpGs had absolute methylation differences greater than 0.05. These included cg06750897 annotated to *PBX1* (Δβ = 0.110, *p*-value = 1.70 × 10^−5^), cg23731272 annotated to *SMAD3* (Δβ = 0.111, *p*-value = 4.31 × 10^−5^), cg02486855 annotated to *SMAD3* (Δβ = 0.109, *p*-value = 9.45 × 10^−5^), and two unannotated CpGs, cg09225388 (Δβ = −0.061, *p*-value = 3.03 × 10^−5^) and cg22789982 (Δβ = 0.103, *p*-value = 3.66 × 10^−5^). DNAm beta-value distributions for the 24 nominally associated CpGs are shown by AGA/LGA status in Figure 3.

To evaluate potential confounding by maternal BMI, we repeated the EWAS with additional adjustment for maternal BMI category, using 25 kg/m^2^ as the cutoff. The sensitivity analysis identified 34 CpGs with nominal *p*-values < 1.00 × 10^−4^, including five with |Δβ| > 0.05; none remained statistically significant after FDR correction (Supplementary Table S1). Of the 24 CpGs identified in the primary EWAS, 14 retained nominal *p*-values < 1.00 × 10^−4^ after BMI adjustment (Supplementary Table S2). The *PBX1*-annotated CpG cg06750897 retained a *p*-value < 1.00 × 10^−4^, whereas the two *SMAD3*-annotated CpGs were attenuated to *p*-values of 1.81 × 10^−4^ and 3.46 × 10^−4^. As expected, Δβ values were unchanged because they were calculated from the same unadjusted LGA and AGA group means, whereas the t-statistics and *p*-values reflected the covariate-adjusted models.

**Figure 4:**
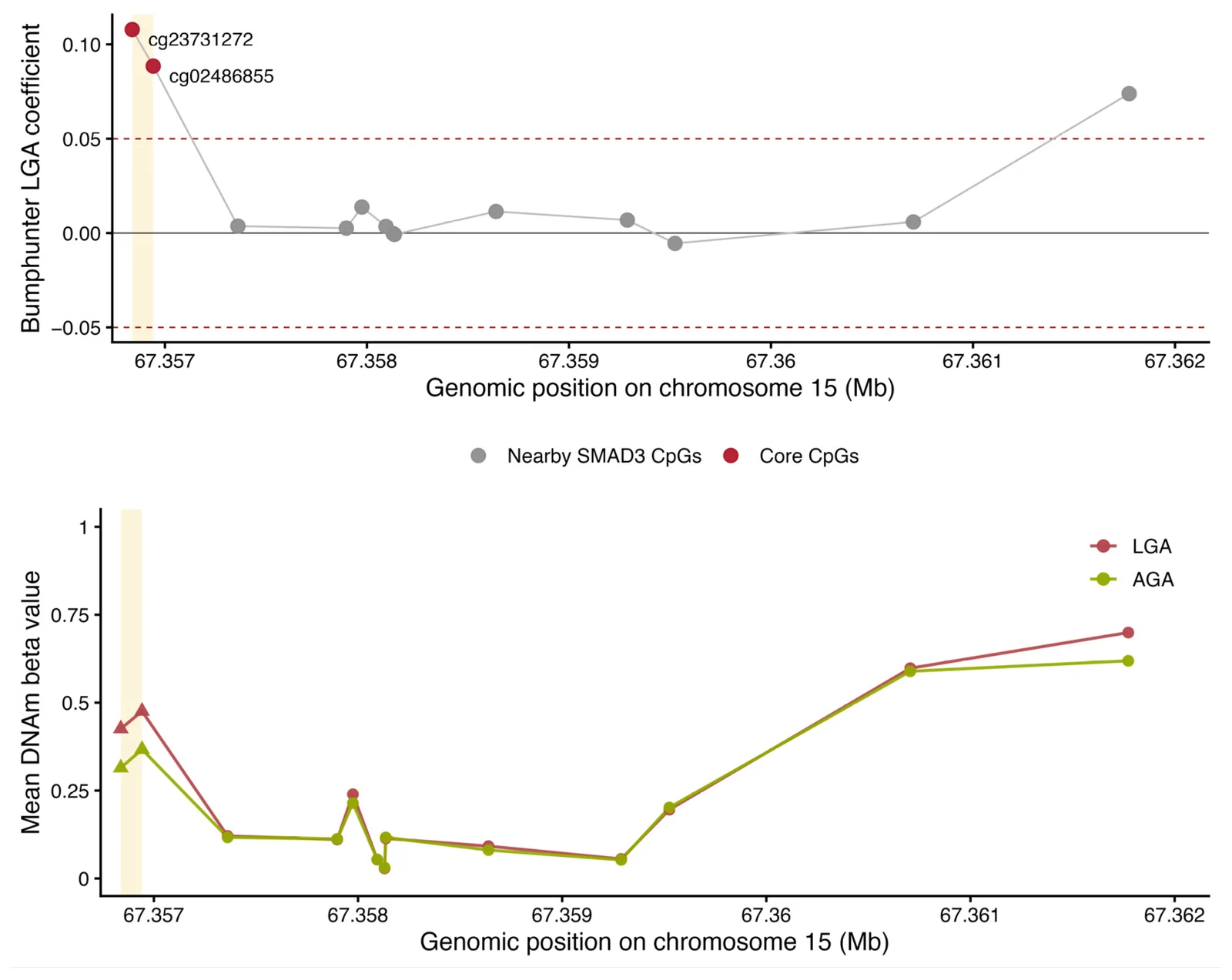
Targeted bumphunter analysis of the SMAD3 locus. The (**upper panel**) shows the adjusted bumphunter coefficient across CpGs in the SMAD3 ± 5 kb window. Dashed lines indicate the ±0.05 coefficient cutoff, and the shaded region indicates the identified two-CpG candidate region; red points indicate the two CpGs in this region. The (**lower panel**) shows mean DNAm beta values for LGA and AGA infants across the same CpGs, with triangle markers indicating the same two candidate CpGs.

Because two CpGs annotated to *SMAD3* were located within 105 bp and showed concordant hypermethylation in LGA infants, we performed an exploratory, targeted bumphunter analysis of the *SMAD3* ± 5 kb window (Figure 4) [27]. Using the same adjusted model and a methylation-effect cutoff of 0.05, bumphunter identified a two-CpG candidate region containing cg23731272 and cg02486855 (chr15:67,356,838–67,356,942), with an average coefficient of 0.098 and an area of 0.196. No bootstrap regions were more extreme across 1000 resamples (family-wise error rates (FWER) < 0.001). Mean DNAm beta values across the same window showed higher methylation in LGA infants at the two candidate CpGs. This targeted result supports *SMAD3* as a candidate locus for follow-up.

## 3. Discussion

This pilot study explored associations between cord blood DNAm and LGA status in the nested LGA case–control sample of the GROW cohort. In the EWAS adjusted for residual PCs and estimated cell type proportions, no CpGs remained statistically significant after FDR correction. However, 24 CpGs showed nominal associations with LGA status, and five appeared to have absolute methylation differences greater than 0.05. Among annotated loci, the strongest signals were in *PBX1* and *SMAD3*, suggesting candidate loci for future evaluation.

*PBX1* (pre-B-cell leukemia homeobox 1) encodes a developmental transcription factor. Notably, *PBX1* was also identified by Agha et al. (2016) in a larger cord blood DNAm study of fetal growth (n = 476) [16]. Agha et al. (2016) [16] identified 34 CpG sites associated with increasing fetal growth based on Z-scores of birth weight for gestational age, including four CpGs annotated to the *PBX1* gene, while the present study examined LGA as a categorical outcome. Despite these differences in study design, the recurrence of *PBX1*-annotated signals across cord blood studies using the Illumina 450K platform (Illumina Inc., San Diego, CA, USA) suggests that this locus may warrant further investigation in studies of fetal overgrowth.

We identified two CpGs located within 105 bp of each other in the promoter-proximal TSS1500 region of *SMAD3* that showed concordant hypermethylation in LGA infants and formed a candidate regional methylation signal. Notably, a previous cord blood study identified increased methylation within the *SMAD3* distal-promoter region, including cg02486855, one of the CpGs identified here, and associated higher neonatal *SMAD3* methylation with increased production of the pro-inflammatory cytokine IL-1β [28]. *SMAD3* is a key mediator of transforming growth factor-beta signaling and has established roles in immune regulation, including regulatory T-cell differentiation and inhibition of macrophage inflammatory responses [29,30,31,32]. Therefore, if the promoter-region hypermethylation observed in our study corresponds to reduced *SMAD3* expression, it could weaken TGF-β-dependent immune regulation and favor a more pro-inflammatory fetal environment. Such a mechanism may be relevant to excessive fetal growth because gestational diabetes and macrosomia have been associated with altered macrophage phenotypes, reduced IL-10, and increased IL-6 and TNF-α [33]. However, this interpretation remains a testable hypothesis because we did not measure *SMAD3* expression, cytokines, or immune-cell function. Because this study used whole cord blood, these findings should be interpreted in the context of leukocyte heterogeneity and immune-related signaling rather than as direct evidence of tissue-specific epigenetic reprogramming. These findings support *SMAD3* as a candidate locus for follow-up, although functional studies are needed to determine whether methylation differences at this locus are associated with gene expression, inflammatory markers, or immune phenotypes.

Prior studies of cord blood DNAm and LGA are limited and differ in sample size, methylation platform, phenotype definition, and analytical approach. Two cord blood studies specifically examining LGA did not identify methylation changes in CpG sites annotated to *PBX1* or *SMAD3*, but both had very small sample sizes and used platforms or approaches different from the Illumina 450K EWAS used here. Carrizosa-Molina et al. included 13 LGA and 12 AGA infants and used the TruSeq Methyl Capture EPIC platform [21], whereas Rondinone et al. included 5 LGA and 5 small-for-gestational-age infants and examined LINE-1 methylation rather than conducting an EWAS [22]. Chiavaroli et al. (2015), which used the Illumina HumanMethylation450 (Illumina Inc., San Diego, CA, USA) array in cord blood, reported no DNAm differences between LGA and AGA infants, although their study design focused on postnatal growth patterns after LGA birth [34].

Although the small sample size with the high-dimensional data necessitates cautious interpretation, these results may motivate future research. Although these annotated loci may provide biologically interpretable candidates for future study, none remained statistically significant after FDR correction. Therefore, these findings should be interpreted as exploratory and hypothesis-generating rather than evidence of confirmed regulatory mechanisms.

There are limitations to our pilot study, mostly related to the high complexity and financial cost of quantifying DNAm. First, while similar to most past studies, the sample size is small, reducing statistical power to detect genome-wide significant associations. After applying FDR correction, there were no genome-wide significant CpGs. Additionally, the cross-sectional design does not establish causation. Information on assisted reproductive technology, including in vitro fertilization (IVF), was not available in the analytic dataset. Therefore, IVF conception status cannot be evaluated as a potential confounder, although prior evidence suggests that assisted reproductive technologies may be associated with offspring DNAm patterns and perinatal growth outcomes [35,36]. The racial homogeneity of the cohort (all cases and controls were white, as described in the Materials and Methods Section) limits generalizability [37]. Exploring the relationship between methylation and LGA across diverse racial/ethnic groups could shed light on potential variations, aiding in understanding the underlying mechanisms and any disparities in health outcomes.

Future studies with larger cohorts and longitudinal designs are necessary to confirm these findings and to explore temporal relationships between maternal factors, DNAm, and fetal growth outcomes. This pilot EWAS of cord blood DNAm did not identify genome-wide significant CpG loci but revealed suggestive methylation differences for sites annotated to two genes, *PBX1* and *SMAD3*. The *PBX1* finding is consistent with results from the largest prior cord blood DNAm study of fetal growth. These findings reinforce emerging evidence that epigenetic correlates of fetal overgrowth in blood are modest in magnitude and likely context-dependent. The results emphasize the importance of replication in larger cohorts using comparable methodology and laboratory platforms, as well as integrative multi-omic approaches. Such studies are needed to clarify whether these methylation differences contribute to, or merely reflect, the intrauterine milieu associated with excessive fetal growth. Our findings add to the limited literature on epigenetic features associated with LGA by providing an initial characterization of cord blood methylation patterns in relation to excessive fetal growth.

## 4. Materials and Methods

### 4.1. Study Cohort and Phenotype Collection

#### 4.1.1. Gestational Regulators of Weight (GROW) Study

The GROW study is a multidisciplinary, longitudinal cohort study of 286 pregnant women initiated to study the relationships between maternal obesity, metabolic factors, and fetal growth. It focused on women who sought early prenatal care at the University of Michigan Health System. Enrollment occurred between six and ten weeks of gestation. Inclusion criteria required participants to be aged between 18 and 45, have a singleton pregnancy, and intend to deliver at the study hospital. Baseline maternal characteristics were collected through a questionnaire administered upon enrollment (between six and ten weeks of gestation) and subsequently verified via review of medical records. Pre-pregnancy BMI was calculated using height and pre-pregnancy weight (BMI = weight/height^2^), and categorized using the BMI distributions defined by the World Health Organization: underweight (BMI < 18.5), normal weight (18.5 ≤ BMI < 25), overweight (25 ≤ BMI < 30), and obese [38]. Additionally, neonatal measures including birth weight, gestational age, and sex were measured at delivery and abstracted from the medical record.

#### 4.1.2. Large for Gestational Age (LGA)

LGA refers to newborn babies who weigh more than the typical amount for their gestational age. It was defined as a birth weight larger than the 90th percentile for gestational week at delivery. In contrast, appropriate-for-gestational-age (AGA) refers to infants whose birth weight falls between the 10th percentile and 90th percentile for their gestational age [38]. Gestational age was determined based on participants’ recall of the first day of their last menstrual period (LMP) prior to 8 weeks’ gestation and corroborated by three independent ultrasonographic measurements of crown-rump length between 6 and 10 weeks’ gestation. If the discrepancy with dates was >6 days, the ultrasound estimate of gestational age was used. Although early first trimester ultrasound-based dating is generally thought to be highly accurate [39], it may be systematically biased in obese individuals [40,41]. This approach aimed to reduce errors from LMP recall by relying on ultrasound estimates [42].

#### 4.1.3. Nested Case–Control Sample

We had funding available for 100 total cases and controls. No formal a priori sample size calculation was performed, as the study was designed as an exploratory EWAS using available archived cord blood DNA samples from the GROW cohort. We restricted selection in this pilot study to white LGA cases as the sample size was too small to control for confounding by race given the extreme distribution of cases by race. There were 36 infants total born LGA in the GROW study: 33 were white, 3 were “other” race, and none were Black or Asian. DNA was extracted for the 33 cases. However, 5 cases failed quality control, leaving 28 LGA cases for DNAm analysis. All cases were born at term. To select AGA controls among the remaining GROW births, we restricted eligibility to AGA births at term to white mothers. We identified 63 AGA infants as controls. For the descriptive analyses, maternal and infant characteristics were summarized by AGA/LGA status. Categorical variables were compared using Fisher’s exact tests. The distributions of continuous variables were evaluated separately within the AGA and LGA groups using histograms, Q-Q plots, and Shapiro–Wilk tests. Approximately normally distributed variables were summarized using the mean and standard deviation and compared using Welch’s two-sample *t*-test. Variables that were not normally distributed were summarized using the median and interquartile range and compared using the Wilcoxon rank-sum test.

### 4.2. DNAm and Data Quality Control

DNAm was measured in genomic DNA isolated from umbilical cord blood by the Michigan Medical Genetics Laboratory using standard procedures and stored at −80 °C. DNAm was measured by the Wayne State University Applied Genomics Technology Center. Laboratory personnel were blinded to birth outcome and other maternal and infant health indicators. Bisulfite-modified DNA was prepared using the EZ-96 DNA Methylation Kit™ according to the manufacturer’s instructions (Zymo Research Corp., Irvine, CA, USA). Quantitative, loci-specific methylation was assessed using the Infinium HumanMethylation450 BeadChip (Illumina Inc., San Diego, CA, USA) per the manufacturer’s instructions. A subset of samples was run in duplicate in order to assess inter-chip variability. CpGenome Universal Methylated DNA was used as a positive control (Millipore, Temecula, CA, USA). Inter-chip variability was assessed, and internal validity was assessed by examining gender-specific methylation of 6 x-linked housekeeping genes (EFNB1, ELK1, FMR1, G6PD, GPC3, GLA) [34,35]. DNAm data preprocessing and quality control were performed in R software using the minfi R package (version 1.18.1; Bioconductor). Raw methylation data were extracted using minfi, and quantile normalization was performed on six separate signal intensity distributions following the 450K preprocessing approach described by Lehne et al. [43]. Data-driven preprocessing and quality-control procedures followed Pidsley et al. [44]. CpG probes were excluded if they failed the detection *p*-value threshold of *p* < 1.00 × 10^−12^, mapped to sex chromosomes, were annotated with a single nucleotide polymorphism within 10 base pairs, or were previously identified as cross-reactive probes. Samples were excluded if they had a call rate < 98% or mismatched predicted and reported sex. After quality control, a total of 428,858 CpGs remained for EWAS.

### 4.3. Epigenome-Wide Association Study on LGA Infants

EWAS was performed to test the association of each CpG methylation with LGA status. We adjusted global confounding factors following a comprehensive analysis pipeline developed by Lehne et al. [43]. Cord blood cell-type proportions were estimated from 450K DNAm data using a reference-based deconvolution approach with the FlowSorted.CordBloodCombined.450k Bioconductor reference dataset [45,46,47]. Estimated proportions included CD8+ T cells, CD4+ T cells, natural killer cells, B cells, monocytes, granulocytes, and nucleated red blood cells. We first regressed DNAm on the potential confounding factors, including age, education level, income, marital status, pregnancy history, diabetes status, alcohol consumption, and exposure to tobacco smoke. We then performed a principal component analysis (PCA) on the resulting regression residuals. The top five principal components ($PC_{1},\ldots .,PC_{5}$) on the residuals were retained and adjusted in the final model. Finally, a generalized linear model was performed for each CpG, with the DNAm beta value as the dependent variable, and LGA status, cell type proportions, and the top five residual PCs as the independent variables. As a sensitivity analysis, we repeated the EWAS with additional adjustment for maternal BMI category using 25 kg/m^2^ as the cutoff. For each CpG site, the association between LGA status and DNAm was assessed using the model-based t statistic and corresponding nominal *p*-value. Δβ was calculated as the mean DNAm beta value in LGA infants minus the mean DNAm beta value in AGA infants; positive values indicate higher methylation in LGA infants, whereas negative values indicate lower methylation in LGA infants compared with AGA infants. CpG sites with nominal *p* < 1.00 × 10^−4^ were considered suggestive associations. FDR correction was applied to account for multiple testing across CpG sites.

### 4.4. Targeted Regional Methylation Analysis

We performed an exploratory targeted regional methylation analysis using the bumphunter R/Bioconductor package to evaluate whether neighboring CpGs showed consistent methylation differences by LGA status [27]. The analysis was conducted using DNAm beta values in a ±5 kb genomic window around the candidate CpGs. The same adjusted model used in the EWAS was applied, including LGA status, the top five residual PCs, and estimated cord blood cell-type proportions. LGA status was specified as the coefficient of interest. Neighboring CpGs were clustered using a maximum gap of 500 bp, and candidate regions were identified using a methylation-effect cutoff of 0.05. The statistical significance of candidate regions was evaluated using 1000 bootstrap resamples to estimate region-level *p*-values and FWER. For visualization, mean DNAm beta values were calculated at each CpG site by averaging beta values across infants within the LGA and AGA groups separately.

## 5. Conclusions

In this pilot EWAS of 28 LGA and 63 AGA infants, no CpG sites reached genome-wide significance after FDR correction. After adjustment for estimated cord blood cell-type proportions, 24 CpGs showed nominal associations with LGA status at *p*-values < 1.00 × 10^−4^, and 5 had absolute Δβ values greater than 0.05. Among annotated loci, the most interpretable signals were observed for PBX1 and SMAD3. The PBX1 finding is consistent with prior evidence linking this locus to fetal overgrowth, whereas two nearby SMAD3 CpGs showed concordant hypermethylation and formed a candidate regional methylation signal. Overall, these results suggest that LGA-associated cord blood DNAm differences, if present, are modest in magnitude but may include biologically interpretable loci related to fetal growth and immune regulation. These findings should be interpreted as exploratory and hypothesis-generating, and larger cell-type-aware studies with independent validation are needed to determine whether these methylation patterns are reproducibly associated with fetal overgrowth.

## Figures and Tables

**Table 1:** Maternal and infant characteristics by case–control status: appropriate-for-gestational-age (AGA) and large-for-gestational-age (LGA) status.

Characteristic	Level	AGA (N = 63)	LGA (N = 28)	*p*-Value
Maternal age	≤30	28 (44.4%)	12 (42.9%)	1
	>30	35 (55.6%)	16 (57.1%)	
Prenatal smoking	Yes	7 (11.1%)	2 (7.1%)	0.714
	No	54 (85.7%)	26 (92.9%)	
Education level	≤College	37 (58.7%)	18 (64.3%)	0.65
	>College	26 (41.3%)	10 (35.7%)	
Income	≤$80,000	32 (50.8%)	16 (57.1%)	0.652
	>$80,000	31 (49.2%)	12 (42.9%)	
Marital status	Married	51 (81.0%)	24 (85.7%)	0.768
	Unmarried	12 (19.0%)	4 (14.3%)	
Parity	Primipara	18 (28.6%)	7 (25.0%)	0.803
	Multipara	45 (71.4%)	21 (75.0%)	
BMI category	Normal (<25)	36 (57.1%)	7 (25.0%)	0.006
	Overweight or obese (≥25)	27 (42.9%)	21 (75.0%)	
Infant sex	Female	41 (65.1%)	17 (60.7%)	0.814
	Male	22 (34.9%)	11 (39.3%)	
Birth weight, g	Mean (SD)	3362.4 (224.0)	4105.9 (224.3)	<0.001
Gestational age at delivery, weeks	Mean (SD)	39.3 (1.1)	39.4 (1.0)	0.762

Values are presented as n (%) for categorical variables and mean (SD) for continuous variables. *p*-values were calculated using Fisher’s exact test for categorical variables and two-sample *t*-tests for continuous variables. AGA, appropriate for gestational age; LGA, large for gestational age; BMI, body mass index.

**Table 2:** The top epigenome-wide DNA methylation CpGs associated with LGA with *p*-values < 1.00 × 10^−4^.

	Probe	CHR	Position	Gene	Group	Mean Beta LGA	Mean Beta AGA	Δβ	*t*	*p*-Value
1	**cg23731272**	15	67356838	*SMAD3*	TSS1500	0.426	0.315	**0.111**	4.338	4.31 × 10^−5^
2	**cg06750897**	1	164545553	*PBX1*	Body	0.637	0.527	**0.110**	4.588	1.70 × 10^−5^
3	**cg02486855**	15	67356942	*SMAD3*	TSS1500	0.476	0.367	**0.109**	4.120	9.45 × 10^−5^
4	**cg22789982**	14	65068635	*NA*	NA	0.478	0.376	**0.103**	4.382	3.66 × 10^−5^
5	**cg09225388**	14	38072498	*NA*	NA	0.281	0.342	**−0.061**	−4.434	3.03 × 10^−5^
6	cg08047889	6	891199	*NA*	NA	0.735	0.697	0.038	4.112	9.72 × 10^−5^
7	cg22731513	13	111451972	*NA*	NA	0.483	0.449	0.034	4.581	1.75 × 10^−5^
8	cg21553656	3	49569379	*DAG1*	Bod	0.885	0.860	0.025	4.163	8.13 × 10^−5^
9	cg17734624	1	115634681	*NA*	NA	0.353	0.377	−0.025	−4.192	7.32 × 10^−5^
10	cg00167259	1	166590947	*FMO9P*	Body	0.875	0.851	0.023	4.244	6.07 × 10^−5^
11	cg18483404	10	124591732	*CUZD1*	3UTR	0.732	0.710	0.023	4.113	9.68 × 10^−5^
12	cg25621096	1	151032116	*CDC42SE1*; *MLLT11*	5UTR; 1stExon; TSS200	0.155	0.133	0.022	4.420	3.20 × 10^−5^
13	cg04411941	17	7692701	*DNAH2*	Body	0.846	0.827	0.019	4.459	2.77 × 10^−5^
14	cg21443284	1	157490968	*FCRL5*	Body	0.678	0.659	0.019	4.790	7.92 × 10^−6^
15	cg06657888	7	62531318	*NA*	NA	0.508	0.526	−0.018	−4.139	8.85 × 10^−5^
16	cg01540023	13	98950565	*FARP1*	Body	0.909	0.891	0.018	4.342	4.26 × 10^−5^
17	cg15529532	10	15077470	*NA*	NA	0.286	0.268	0.018	4.219	6.64 × 10^−5^
18	cg21995686	4	153917484	*NA*	NA	0.292	0.275	0.017	4.127	9.23 × 10^−5^
19	cg05039241	14	61124922	*NA*	NA	0.094	0.109	−0.014	−4.223	6.54 × 10^−5^
20	cg20161772	1	166846872	*TADA1*	TSS1500	0.869	0.883	−0.014	−4.125	9.30 × 10^−5^
21	cg27234490	3	58174435	*NA*	NA	0.886	0.874	0.012	4.195	7.25 × 10^−5^
22	cg05314310	6	35775304	*LHFPL5*	Body	0.831	0.842	−0.011	−4.407	3.35 × 10^−5^
23	cg03065197	9	139261335	*CARD9*	Body	0.851	0.842	0.009	4.190	7.36 × 10^−5^
24	cg11654620	22	50969039	*TYMP*; *ODF3B*	TSS1500; Body	0.128	0.120	0.008	4.118	9.54 × 10^−5^

Group indicates the genomic region or gene feature to which each CpG site is annotated. Δβ was calculated as the mean DNAm beta value in LGA infants minus the mean DNAm beta value in AGA infants; positive values indicate higher methylation in LGA infants, whereas negative values indicate lower methylation in LGA infants compared with AGA infants. The table is ordered by absolute Δβ. Bolded CpGs indicate sites with |Δβ| > 0.05. The t value represents the test statistic from the epigenome-wide association model. The model included LGA status, the top five residual principal components, and estimated cord blood cell-type proportions. *p*-values are nominal, unadjusted *p*-values. No CpG sites remained statistically significant after false discovery rate correction.

## Data Availability

The data presented in this study are available on request from the corresponding author due to restrictions related to participant privacy and informed consent, which did not permit public release of epigenetic data for outside investigator analysis. Requests for access are subject to approval by the study investigators and any required institutional review board approval.

## Supplementary Materials

The following supporting information can be downloaded at https://www.mdpi.com/article/10.3390/epigenomes10030051/s1. Figure S1. Distributional assessment of birth weight and gestational age at delivery by AGA and LGA status. Table S1. The top epigenome-wide DNA methylation CpGs associated with LGA with *p*-value < 1.00 × 10^−4^ after additional adjustment for maternal BMI ≥ 25. Table S2. Comparison of the 24 CpGs in primary Table 2 before and after additional adjustment for maternal BMI ≥ 25.

## Abbreviations

The following abbreviations are used in this manuscript:

GROW	Gestational Regulators of Weight cohort
LGA	Large for Gestational-Age
AGA	Appropriate for Gestational-Age
DNAm	DNA Methylation
CpG	Cytosine-Phosphate-Guanine
EWAS	Epigenome-Wide Association Study
FDR	False Discovery Rate
