# Supplementary material for: Cord Blood DNA Methylation and Large-for-Gestational-Age Birth: A Pilot Epigenome-Wide Study in the GROW Cohort
Source: Epigenomes. 2026 Aug 4;10(3):51. doi: 10.3390/epigenomes10030051 (PMC13510515; doi:10.3390/epigenomes10030051)
Supplement: Supplementary file 1 [file epigenomes-10-00051-s001.zip › epigenomes-4330096-supplementary.pdf]

Supplementary Material

Cord Blood DNA Methylation and Large-for-Gestational-Age Birth: A Pilot Epigenome-Wide Study in the GROW Cohort

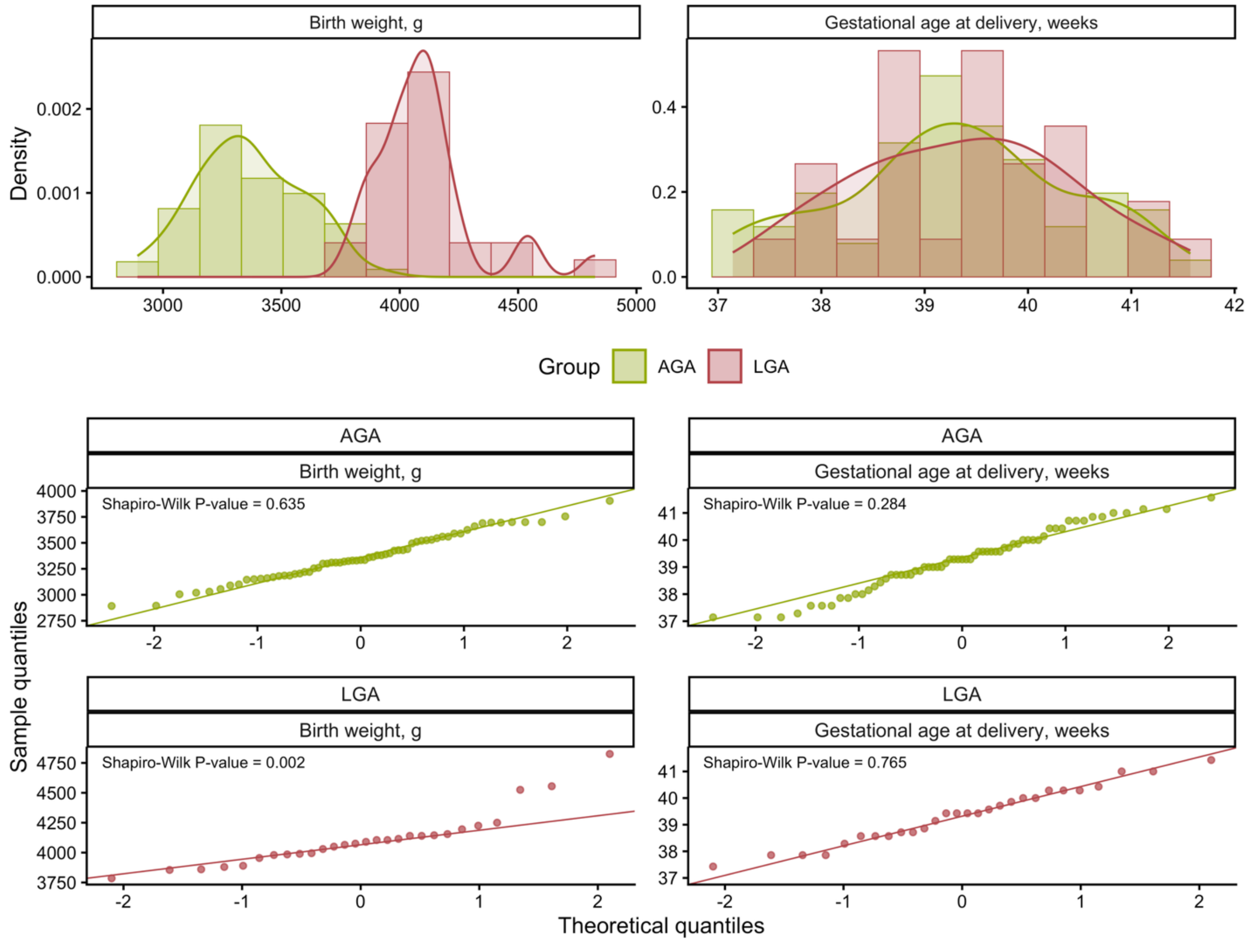

Figure S1. Distributional assessment of birth weight and gestational age at delivery by AGA and LGA status. The upper panels show histograms with density curves, and the lower panels show Q-Q plots with group-specific Shapiro-Wilk P-values. Birth weight departed from normality in the LGA group, whereas gestational age showed no evidence of departure from normality in either group.

Table S1. The top epigenome-wide DNA methylation CpGs associated with LGA with P-value<1.00E-04 after additional adjustment for maternal BMI ≥25.

Probe	CHR	Position	Gene	Group	Mean beta LGA	Mean beta AGA	Δβ	t	P-value
**cg06750897**	**1**	**164545553**	***PBX1***	**Body**	**0.637**	**0.527**	**0.110**	**4.217**	**6.84E-05**
**cg22789982**	**14**	**65068635**			**0.478**	**0.376**	**0.103**	**4.766**	**9.00E-06**
**cg22744079**	**4**	**155410860**	***DCHS2***	**1stExon**	**0.539**	**0.618**	**-0.078**	**-4.185**	**7.68E-05**
**cg12580503**	**12**	**130340287**	***TMEM132D***	**Body**	**0.858**	**0.794**	**0.063**	**4.331**	**4.53E-05**
**cg09225388**	**14**	**38072498**			**0.281**	**0.342**	**-0.061**	**-4.188**	**7.58E-05**
cg22731513	13	111451972			0.483	0.449	0.034	4.553	2.01E-05
cg17182240	11	126768919	*KIRREL3*	Body	0.717	0.746	-0.029	-4.170	8.08E-05
cg25819879	2	3622620	*RPS7*	TSS1500	0.215	0.243	-0.029	-4.194	7.44E-05
cg20442322	2	84743743	*DNAH6*	5UTR	0.181	0.209	-0.028	-4.238	6.35E-05
cg21553656	3	49569379	*DAG1*	Body	0.885	0.860	0.025	4.203	7.20E-05
cg17734624	1	115634681			0.353	0.377	-0.025	-4.133	9.22E-05
cg25621096	1	151032116	*CDC42SE1; MLLT11*	5UTR; 1stExon; TSS200	0.155	0.133	0.022	4.269	5.68E-05
cg21792134	3	121491382	*IQCB1*	Body	0.565	0.585	-0.020	-4.120	9.66E-05
cg04411941	17	7692701	*DNAH2*	Body	0.846	0.827	0.019	4.124	9.53E-05
cg10764440	12	4489221	*FGF23*	TSS1500	0.733	0.714	0.019	4.222	6.72E-05
cg21443284	1	157490968	*FCRL5*	Body	0.678	0.659	0.019	5.017	3.43E-06
cg16622651	6	76724431	*IMPG1*	Body	0.714	0.732	-0.019	-4.384	3.75E-05
cg06657888	7	62531318			0.508	0.526	-0.018	-4.273	5.60E-05
cg07005294	8	23572029			0.074	0.090	-0.016	-4.163	8.28E-05
cg06219806	11	62308810	*AHNAK*	5UTR	0.787	0.772	0.015	4.245	6.20E-05
cg11430353	2	174881312			0.765	0.780	-0.015	-4.190	7.54E-05
cg05039241	14	61124922			0.094	0.109	-0.014	-4.655	1.37E-05
cg19928703	13	30143971	*SLC7A1*	5UTR	0.768	0.783	-0.014	-4.123	9.57E-05
cg19577548	20	48178928	*PTGIS*	Body	0.723	0.738	-0.014	-4.332	4.51E-05
cg20161772	1	166846872	*TADA1*	TSS1500	0.869	0.883	-0.014	-4.347	4.28E-05
cg06183559	13	95357818			0.156	0.170	-0.014	-4.309	4.92E-05
cg22668906	11	128180077			0.746	0.760	-0.014	-4.429	3.18E-05
cg10904854	3	185742220			0.840	0.854	-0.014	-4.113	9.90E-05
cg00974685	10	113759413			0.729	0.741	-0.012	-4.626	1.53E-05

cg05314310	6	35775304	*LHFPL5*	Body	0.831	0.842	-0.011	-4.197	7.34E-05
cg09044785	20	30458634	*TTLL9; DUSP15*	5UTR; TSS1500; TSS200; 1stExon	0.115	0.105	0.010	4.153	8.59E-05
cg03065197	9	139261335	*CARD9*	Body	0.851	0.842	0.009	4.404	3.47E-05
cg27230749	1	20123989	*TMCO4*	5UTR	0.843	0.835	0.008	4.143	8.91E-05
cg00113315	1	9098035	*SLC2A5*	Body	0.778	0.786	-0.008	-4.214	6.91E-05

Group indicates the genomic region or gene feature to which each CpG site is annotated. Δβ was calculated as the mean DNAm beta value in LGA infants minus the mean DNAm beta value in AGA infants; positive values indicate higher methylation in LGA infants, whereas negative values indicate lower methylation in LGA infants compared with AGA infants. The table is ordered by absolute Δβ. Bolded CpGs indicate sites with |Δβ| > 0.05. The t value represents the test statistic from the epigenome-wide association model. The model included LGA status, the top five residual principal components, estimated cord blood cell-type proportions, and maternal BMI. P-values are nominal, unadjusted P-values. No CpG sites remained statistically significant after false discovery rate correction.

Table S2. Comparison of the 24 CpGs in primary Table 2 before and after additional adjustment for maternal BMI ≥25

Probe	CHR	Position	Gene	Group	Δβ	t (original model)	t (new model)	P-value (original model)	P-value (new model)
**cg23731272**	15	67356838	*SMAD3*	TSS1500	0.111	4.338	3.941	**4.31E-05**	1.81E-04
**cg06750897**	1	164545553	*PBX1*	Body	0.110	4.588	4.217	**1.70E-05**	**6.84E-05**
**cg02486855**	15	67356942	*SMAD3*	TSS1500	0.109	4.120	3.750	**9.45E-05**	3.46E-04
**cg22789982**	14	65068635			0.103	4.382	4.766	**3.66E-05**	**9.00E-06**
**cg09225388**	14	38072498			-0.061	-4.434	-4.188	**3.03E-05**	**7.58E-05**
cg08047889	6	89116499			0.038	4.112	3.875	**9.72E-05**	2.26E-04
cg22731513	13	111451972			0.034	4.581	4.553	**1.75E-05**	**2.01E-05**
cg21553656	3	49569379	*DAG1*	Body	0.025	4.163	4.203	**8.13E-05**	**7.20E-05**
cg17734624	1	115634681			-0.025	-4.192	-4.133	**7.32E-05**	**9.22E-05**
cg00167259	1	166590947	*FMO9P*	Body	0.023	4.244	3.926	**6.07E-05**	1.90E-04
cg18483404	10	124591732	*CUZD1*	3UTR	0.023	4.113	3.552	**9.68E-05**	6.65E-04
cg25621096	1	151032116	*CDC42SE1; MLLT11*	5UTR; 1stExon; TSS200	0.022	4.420	4.269	**3.20E-05**	**5.68E-05**
cg04411941	17	7692701	*DNAH2*	Body	0.019	4.459	4.124	**2.77E-05**	**9.53E-05**
cg21443284	1	157490968	*FCRL5*	Body	0.019	4.790	5.017	**7.92E-06**	**3.43E-06**
cg06657888	7	62531318			-0.018	-4.139	-4.273	**8.85E-05**	**5.60E-05**
cg01540023	13	98950565	*FARP1*	Body	0.018	4.342	3.895	**4.26E-05**	2.12E-04
cg15529532	10	15077470			0.018	4.219	4.074	**6.64E-05**	1.14E-04
cg21995686	4	153917484			0.017	4.127	3.806	**9.23E-05**	2.86E-04
cg05039241	14	61124922			-0.014	-4.223	-4.655	**6.54E-05**	**1.37E-05**
cg20161772	1	166846872	*TADA1*	TSS1500	-0.014	-4.125	-4.347	**9.30E-05**	**4.28E-05**
cg27234490	3	58174435			0.012	4.195	4.029	**7.25E-05**	1.33E-04
cg05314310	6	35775304	*LHFPL5*	Body	-0.011	-4.407	-4.197	**3.35E-05**	**7.34E-05**
cg03065197	9	139261335	*CARD9*	Body	0.009	4.190	4.404	**7.36E-05**	**3.47E-05**
cg11654620	22	50969039	*TYMP; ODF3B*	TSS1500; Body	0.008	4.118	3.819	**9.54E-05**	2.74E-04

Group indicates the genomic region or gene feature to which each CpG site is annotated. Δβ was calculated as the mean DNAm beta value in LGA infants minus the mean DNAm beta value in AGA infants. The original model refers to the primary EWAS, and the new model includes additional adjustment for maternal BMI. Nominal P-values <1.00E-04 are shown in bold. Bolded CpGs indicate sites with |Δβ| > 0.05.
